# Silymarin ameliorates diazinon-induced subacute nephrotoxicity in rats via the Keap1–Nrf2/heme oxygenase-1 signaling pathway

**DOI:** 10.1007/s11419-024-00697-x

**Published:** 2024-08-08

**Authors:** Eman Mohamed Fath, Hatem H. Bakery, Ragab M. EL-Shawarby, Mohamed E. S. Abosalem, Samar S. Ibrahim, Nesrine Ebrahim, Ahmed Medhat Hegazy

**Affiliations:** 1 Department of Forensic Medicine and Toxicology, Faculty of Veterinary Medicine, Benha University, Moshtohor, Toukh, 13736 Qalyubia Egypt; 2 Department of Histology and Cell Biology, Faculty of Medicine, Benha University, Benha, 13511 Egypt; 3 Stem Cell Unit, Faculty of Medicine, Benha University, Benha, 13511 Egypt; 4 Faculty of Medicine, Benha National University, Obour City, Egypt

**Keywords:** Diazinon, Heme oxygenase-1, Keap1, Nephrotoxicity, NF-κB, Silymarin

## Abstract

**Purpose:**

The goal of the current study was to clarify the potential molecular mechanism underlying the protective effects of silymarin (SIL) administration against diazinon-induced subacute nephrotoxicity, with a special emphasis on the role of the Kelch-like-associated protein-1 (Keap1)–nuclear factor erythroid 2-related factor 2 (Nrf2)–heme oxygenase-1 (HO-1) signaling pathway in minimizing the oxidative stress induced by diazinon (DZN).

**Methods:**

Five equal groups of thirty adult male Wistar rats were created at random. Group 1 (G1) was maintained under typical control conditions and administered saline intragastrically (I/G) once daily for 4 weeks; G2 was administered olive oil I/G for 4 weeks; G3 was I/G administered silymarin daily for 4 weeks; G4 was I/G administered diazinon daily for 4 weeks. G5 was I/G administered silymarin daily 1 h before the I/G administration of the diazinon for 4 weeks. Blood samples were collected at the end of the experiment for the determination of complete blood cell count, and kidney function tests. Kidney specimens were collected for the evaluation of the oxidative markers, mRNA gene expression, protein markers, and histopathological examination.

**Results:**

SIL reduced the renal dysfunction caused by DZN by restoring urea and creatinine levels, as well as oxidative indicators. Although the expression of Keap-1 was also elevated, overexpression of Nrf2 also enhanced the expression of HO-1, a crucial target enzyme of Nrf2.

**Conclusions:**

SIL is hypothesized to potentially aid in the prevention and management of nephrotoxicity caused by DZN.

**Supplementary Information:**

The online version contains supplementary material available at 10.1007/s11419-024-00697-x.

## Introduction

Pesticides are widely utilized across the world to increase food production, protect human and animal health, and manage household, veterinary, and agricultural insect pests and disease vectors [1]. Pesticide poisoning causes an estimated 3 million cases of poisoning annually, more than 200,000 of which result in death [2]. An organophosphorus insecticide known as diazinon (DZN) [phosphoric acid, *O*,*O*-diethyl *O*-(2-isopropyl-6-methyl-4-pyridinyl) phosphorothioate] is utilized on a global scale [3]. According to a previous study [4], comment on inhibition of cholinesterase activity, exposure to DZN can cause major histopathological lesions in a variety of tissues, including the kidney, brain, and liver. Many of these effects are caused by excess free radical generation, the consumption of tissue antioxidants, and oxidative stress [5]. Previous investigations have clearly shown that the nephrotoxicity of DZN is caused by oxidative damage [6]. *Silybum marianum* (family Asteraceae) produces an extract called silymarin (SIL) from its seeds [7]. It has been used as a natural remedy for many years, primarily to treat liver conditions. In preclinical tests, SIL, a milk thistle bioactive extract, has shown antioxidant and hepatoprotective effects [8]. SIL, a blend of flavonolignans derived from milk thistle (*Silybum marianum* Gaertneri), contains silibinin as its primary component, along with silydianin, silycristin, isosilybin A, isosilybin B, isosilycristin, and taxifolin. According to Ferenci [9], silibinin exhibits substantial antioxidant, antifibrotic, and antiapoptotic effects that make it a possible therapeutic option for hepatic diseases. The capability of SIL to decrease lipid peroxidation and enhance glutathione is one of the factors contributing to its hepatoprotective effects. It also has high detoxifying and antioxidant activities [10]. The renal effects of silymarin are less well known than its hepatic effects. In ischemia‒reperfusion and medication nephrotoxicity models in rats, silymarin has been shown to have beneficial effects in several studies [11]. Studies examining the hepatic effects of silymarin have provided information on its effects on renal function. Few studies have analyzed renal histology, despite reports that renal function has not declined. Its kidney-protective impact should be assessed in light of its potent antioxidant and hepatoprotective effects. The present study focused on how silymarin inhibited oxidative stress via the Kelch-like-associated protein-1 (Keap1)–nuclear factor erythroid 2-related factor 2 (Nrf2)–heme oxygenase-1 (HO-1) signaling pathway to investigate its effectiveness against DZN-induced nephrotoxicity.

## Materials and methods

### Chemicals

We purchased DZN 60% (emulsifiable concentrate) from the High Control Company (Cairo, Egypt) which included 60% active component. DZN was dissolved in olive oil to obtain the necessary concentration. SIL (> 45%) was obtained from Medical Union Pharmaceuticals in Abu-Sultan, Ismailia, Egypt. Prior to being administered to the rats, the dose of each chemical was adjusted.

### Experimental animals

Three-month-old male adult Wistar rats (weighing 200–250 g) were purchased from the Faculty of Veterinary Medicine, Experimental Animal Unit, Benha University, Egypt. Rats were housed in orderly cages and were provided with clean food and water on demand. All rats were housed under standard conditions, including a room temperature of 23 °C, a 12-h light/dark cycle, and free access to water and food. Prior to therapy, baseline body weight measurements were recorded for all groups. Animal weights were recorded weekly to modify the chemical dosage.

### Experimental design

Five equal groups of six adult male Wistar rats each were created from 30 rats. The initial group (G1) was maintained as a healthy control group and was intragastrically (I/G) administered 0.1 mL of saline once daily for 4 weeks. The second group (G2) was I/G administered 0.1 mL of olive oil once daily for 4 weeks. The third group (G3) was I/G administered SIL dissolved in an aqueous solution at a dose of 200 mg/kg body weight [12], daily for 4 weeks. The fourth group (G4) was I/G administered DZN dissolved in olive oil at a dose of 15 mg/kg body weight [13] daily for 4 weeks. The fifth group (G5) was administered SIL 1 h before the I/G administration of DZN at the same dose and duration used in G3 and G4. The experimental rats were anesthetized with isoflurane after the study was complete before being euthanized by decapitation, after which blood samples were collected for the estimations of the complete blood count, urea levels, creatinine levels, levels of oxidative/antioxidant markers, gene expression, and histopathological changes in the kidney.

### Preparation of kidney homogenates

Kidney tissue homogenates were prepared according to the methods of El-Shaer et al. [14]. The oxidative indicators [catalase (CAT) activity, superoxide dismutase (SOD) activity, glutathione peroxidase (GSH-Px) level, reduced glutathione (GSH) level, and level of the lipid peroxidation byproduct MDA], IL-1β, IL-6, and total protein contents were measured for the collected supernatant.

### Assay methods

#### Complete blood cell count

According to Barber et al. [15], automatic cell counters (Celltac Alpha VET MEK-6550, Nihon Kohden Europe—Nihon Kohden Corporation, Japan), which rely on both electrical and optical approaches, were used to evaluate the complete blood cell counts in all obtained blood samples.

#### Kidney function tests

Serum urea and creatinine concentrations were measured using the methods of Skeggs [16] and Weissman et al. [17], respectively.

### Oxidative markers in kidney tissue homogenates

The levels of catalase (CAT), superoxide dismutase (SOD), glutathione peroxidase (GSH-Px), reduced glutathione (GSH) and malondialdehyde (MDA) were determined using commercially available ELISA kits (BioVision Inc., 155 S. Milpitas Blvd, Milpitas, CA, 95035, USA). Based on the Bradford [18] method, the protein content of the tissue homogenate was measured using a protein estimation ELISA kit (Genei, Bangalore). An enzyme-linked immunosorbent assay (ELISA) plate reader (Stat Fax 2200, Awareness Technologies, Florida, USA) was used to measure color absorbance.

### Cytokines in kidney tissue homogenates

The levels of interleukin-1β (IL-1β) (SEA563Ra) and interleukin-6 (IL-6) (SEA079Ra) were determined using commercially available ELISA kits (Cloud-Clone Corp Co., Houston, USA) according to the manufacturer’s instructions. The protein concentrations are displayed as pg/mL.

### Kidney mRNA gene expression

Real-time polymerase chain reaction (PCR) was used to assess the mRNA expression of the nuclear factor kappa B (NFκB), Kelch-like ECH-associated protein-1 (Keap1), heme oxygenase-1 (HO-1), and nuclear factor erythroid 2-related factor 2 (Nrf2) genes in the kidney. Primer sets for the following genes were utilized: NFκB, Keap-1, HO-1, Nrf2, and GAPDH (Supplementary Table S1). Thermal cycling and fluorescence detection were performed using an Applied Biosystems 7300 real-time PCR system (Foster City, California, USA). Cycle threshold (Ct) values acquired from real-time PCR equipment were applied to a reference (housekeeping) gene (GAPDH) to detect changes in gene expression [19].

### Protein markers in kidney tissue homogenates

We used a ReadyPrep™ protein extraction kit from Bio-Rad Inc. (catalog #163–2086) to extract proteins from kidney tissues. Protein concentrations were measured using the Bradford Protein Assay Kit (SK3041) from Bio Basic Inc. (Markham, Ontario, L3R 8T4 Canada). The quantity of the protein samples loaded in each well was 20 μg. The extracts were separated by sodium dodecyl sulfate–polyacrylamide gel electrophoresis (SDS-PAGE) (12%) and then transferred to polyvinylidene fluoride (PVDF) membranes (Bio-Rad) (7 min at 25 V). The separation was done by a Cleaver electrophoresis unit (Cleaver, UK). After the membrane was blocked for 1 h at room temperature with Tris-buffered saline containing Tween 20 (TBST) (pH 7.6) and 3% bovine serum albumin (BSA), it was incubated with a variety of primary antibodies. Primary antibodies against NFκB, Keap-1, HO-1, Nrf2, and β-actin were purchased from OriGene Technologies, Inc. (9620 Medical Center Drive, Ste 200 Rockville, MD 20850, USA). Then, the membranes were incubated with primary antibodies (anti-NFκB, anti-HO-1, anti-Keap-1, anti-Nrf2, and anti-β actin (housekeeping protein)) at 4 °C overnight. The blot was rinsed with TBST 3–5 times for 5 min. The target proteins on the membrane were treated with the goat anti-rabbit IgG-HRP-1 mg goat mab-Novus Biologicals secondary antibody solution for 1 h at room temperature. Next, the blot was washed three times for 5 min using TBST. The blot was covered with a chemiluminescent substrate (catalog no. 170–5060, Clarity™ Western ECL substrate; Bio-Rad). The chemiluminescent signals were captured using an imager based on a CCD camera (Chemi Doc imager, Biorad, USA). After protein normalization on the imager, the band intensity of the target proteins was compared with the band intensity of the control, β-actin (housekeeping protein).

### Histopathological examination

In all groups, kidney samples were collected immediately and then fixed for 24 h in 10% buffered neutral formalin. Washing was performed using tap water; then, serial dilutions of alcohol (methyl, ethyl and absolute ethyl) were used for dehydration. Specimens were cleared in xylol, embedded in paraffin, dehydrated in various grades of ethyl alcohol. Paraffin bees-wax tissue blocks were prepared for sectioning at 4 µm thickness using RM2235 Leica microtome (Biosystems, Nussloch, Germany). Hematoxylin and eosin (H&E) and Masson’s trichrome staining and a microscopic examination were performed [20].

### Immunohistochemical study

Deparaffinized and hydrated paraffin sections were used. The sections were first blocked for nonspecific reactions with 10% hydrogen peroxide, followed by an incubation with the buffer solution containing primary rabbit polyclonal antibodies against cytochrome C (SAB4502234; 1:50–1:100 dilution; Sigma‒Aldrich, St. Louis, Missouri, USA), rabbit monoclonal antibodies against cyclooxygenase 2 (COX2) ([EPR12012] (ab179800); 1/1000–1/5000 dilution; Abcam, UK), and a mouse monoclonal antibody against tumor necrosis factor-α (TNF-α) [TNFA/1172] (ab220210) at a concentration of 2–4 µg/mL. Before beginning the IHC staining with a rabbit polyclonal antibody against SERCA2 ATPase (ab3625, Abcam, UK, 1 g/mL), heat-mediated antigen retrieval was performed with citrate buffer (pH 6). Next, a biotinylated goat anti-rabbit secondary antibody was applied after the cells were washed with phosphate buffer. Sections were treated with labeled avidin–biotin peroxidase, which binds to the biotin on the secondary antibody, to localize the immunological response. Diaminobenzidine was used as a chromogen to visualize the location where the antibody bound because peroxidase transformed it to a brown precipitate [21].

### Morphometric study

Using the Image-Pro Plus program version 6.0 (Media Cybernetics Inc., Bethesda, Maryland, USA), the mean area percentage (area%) of collagen fiber deposition and TNF- and COX2 immunoexpression were calculated. The mean area% for each marker was determined by averaging the data from five photographs taken from five different fields for each rat in each group.

### Statistical analysis

The statistical analysis was performed using SPSS for Windows (Version 18.0; SPSS Inc., Chicago, Illinois) statistical software for social research. With Duncan’s multiple range test and post hoc analysis, one-way ANOVA was utilized to identify significant differences between experimental groups. The means and standard errors of the means (SEMs) were calculated to report the results. *P* values less than 0.05 were regarded as significant.

## Results

Throughout the study period, there were no recorded deaths or symptoms among the different treatment groups.

### Complete blood cell count

The means and standard errors of the hematological parameters for the various groups are shown in Supplementary Table S2. The data revealed that compared with the other treatments, DZN significantly reduced RBC counts, the Hb content, and PCV percentages. Additionally, significant reductions in the platelet count were observed in the DZN-treated group compared with the SIL-pretreated group, and this reduction was reversed in the fifth group when the rats were treated with both DZN and SIL. On the other hand, compared with the other experimental groups, the DZN-treated group showed a significant increase in WBC count. This increase in WBC count was attributed to a significant increase in the absolute numbers of lymphocytes, neutrophils and eosinophils. However, after 4 weeks of treatment, the number of WBCs decreased considerably in the G5 which were rats treated with SIL and then with DZN.

### Serum urea and creatinine levels

Supplementary Fig. S1 displays the means and standard errors for the serum urea and creatinine levels in the various groups. Compared with those in the control and olive oil groups, urea and creatinine levels increased noticeably in the G4 group after 4 weeks of the experiment. However, rats cotreated with both SIL and DZN (G5) had significantly lower serum urea and creatinine levels than the DZN group.

### Changes in kidney oxidative markers

Supplementary Table S3 shows the means and standard errors of the oxidative markers in the individual groups. Rats (G2) which were treated with DZN had significantly higher MDA levels in their kidney tissues than the rats in the control and olive oil groups. Meanwhile, treatment of rats with both DZN and SIL (G5) significantly decreased the MDA level compared with that in the toxin group after 4 weeks of the experiment. Similarly, the levels of CAT, SOD, GPx, and GSH in the kidney tissues from the rats (G4) which were treated with the toxin DZN levels were markedly lower than those in the control and olive oil groups. However, treatment of the toxin-treated rats with SIL (G5) resulted in a considerable increase in the CAT, SOD, GPx, and GSH levels compared with those in the toxin-treated group (G4) after 4 weeks of the experiment.

### Changes in cytokine levels in kidney tissue homogenates

As displayed in Supplementary Fig. S2, rats (G4) that were treated with DZN had noticeably increased concentrations of IL-6 and IL-1β compared to the control and olive oil groups after 4 weeks of the experiment. However, rats which were cotreated with both SIL and DZN (G5) had significantly lower concentrations of IL-6 and IL-1β than those in the toxin-treated group (G4).

### NF-κB, keap-1, HO-1, and Nrf2 mRNA expression in kidney tissue

Supplementary Fig. S3 shows the expression of the NF-κB, keap-1, HO-1, and Nrf2 mRNAs in the kidneys of the control, olive oil-, SIL-, DZN-, and SIL/DZN-treated groups after 4 weeks of treatment. Kidney keap-1, HO-1, and Nrf2 levels were markedly downregulated in the intoxicated rats, and the NF-κB mRNA was markedly upregulated in the kidney. However, treatment of intoxicated rats with SIL markedly upregulated the keap-1, HO-1, and Nrf2 mRNAs and markedly downregulated the NF-κB mRNA in the kidney.

### Changes in protein markers in the kidney

**Fig. 1:**
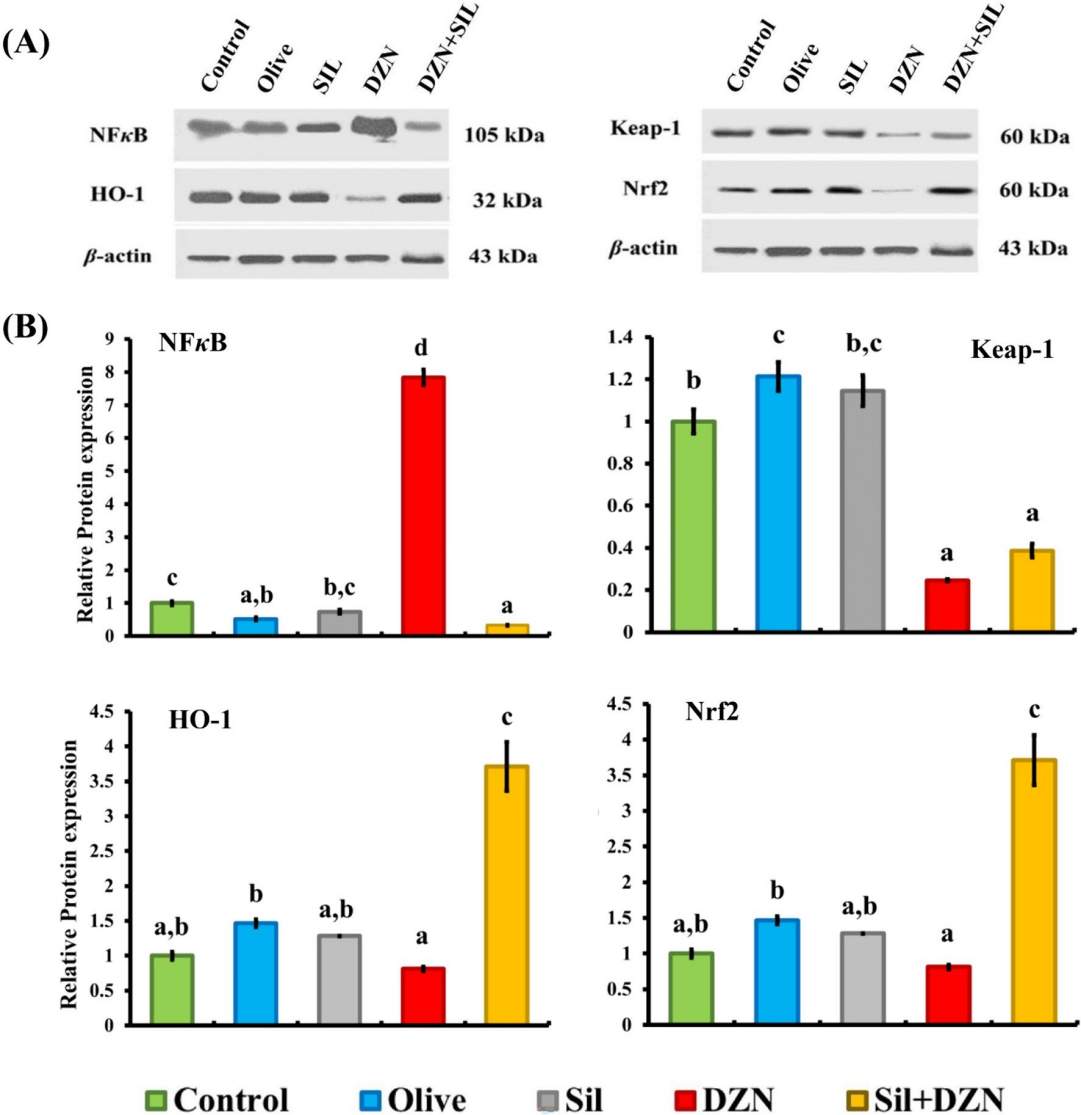
Western blots with densitometric analysis of the Nrf2, keap-1, HO-1, and NF-κB proteins in kidney tissue homogenates from rats treated with olive oil, silymarin, diazinon, silymarin plus diazinon, and the control at 4 weeks after treatment (*n* = 6)

Figure 1 shows that the average relative density of NF-κB, Keap1, HO-1, and Nrf2 normalized to that of GAPDH in kidneys from the SIL-, DZN-, and SIL–DZN-treated groups was greater comparing with that in the control and olive oil-treated groups after 4 weeks of the experiment. Compared with those in the control and olive oil groups, noticeably higher levels of NF-κB (p65) and a substantial decrease in the protein expression of Nrf2, Keap1, and HO-1 were observed in kidney tissues from the intoxicated group (G4). After 4 weeks of the experiment, the rats in the intoxicated group that received SIL treatment (G5) had significantly lower levels of NF-κB (p65) and significantly higher levels of Nrf2, Keap1, and HO-1 than those in the intoxicated group (G4).

### Histopathological assessment of kidney tissue

**Fig. 2:**
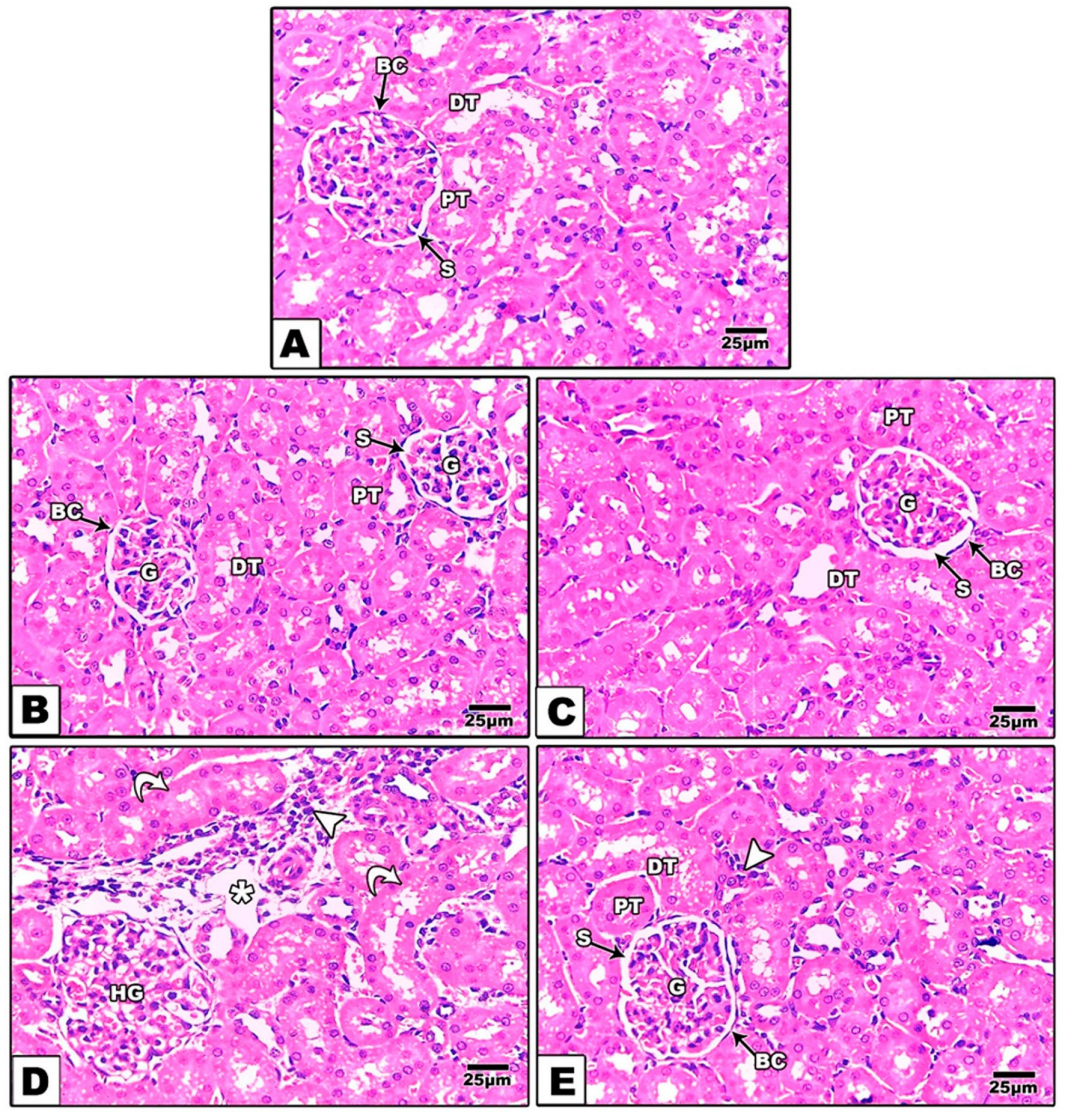
Photomicrograph of the kidneys of the experimental rats. **A**–**C** Normal control rats (G1), olive oil-treated rats (G2) and rats treated with silymarin (G3). The kidney showed a normal glomerulus (G) and Bowmen’s capsule, distal tubules (DTs) and proximal tubules (PTs). **D** Intoxicated rats (G4) induced by diazinon showed dilatation of Bowman’s space and degenerated glomeruli (DG) and inflammatory cell infiltration (arrow), as well as glomerular and peritubular vascular congestion and swelling of renal tubular epithelial cells (curved arrow). **E** Intoxicated rats treated with diazinon and then treated with silymarin (G5) had normal glomeruli (G) and tubules (PTs & DTs). H&E, ×200; the scale bar represents 25 μm

The examination of kidney sections revealed that intoxicated rats (G4) had a dilated Bowman’s space, inflammatory cell infiltration, glomerular and peritubular vascular congestion and swelling of renal tubular epithelial cells (Fig. 2D). Cotreatment of intoxicated rats with SIL resulted in a nearly normal histological structure, as Bowman’s space dilatation, inflammatory cell infiltration, glomerular and peritubular vascular congestion and renal tubular epithelial cell swelling decreased (Fig. 2E). The kidney tissues of rats in the olive oil, SIL and control groups appeared to be normal; they exhibited renal corpuscles consisting of glomeruli surrounded by a narrow Bowman’s space and Bowman’s capsule. The corpuscles were surrounded by proximal and distal convoluted tubules (Fig. 2A–C).

### Masson’s trichrome staining

**Fig. 3:**
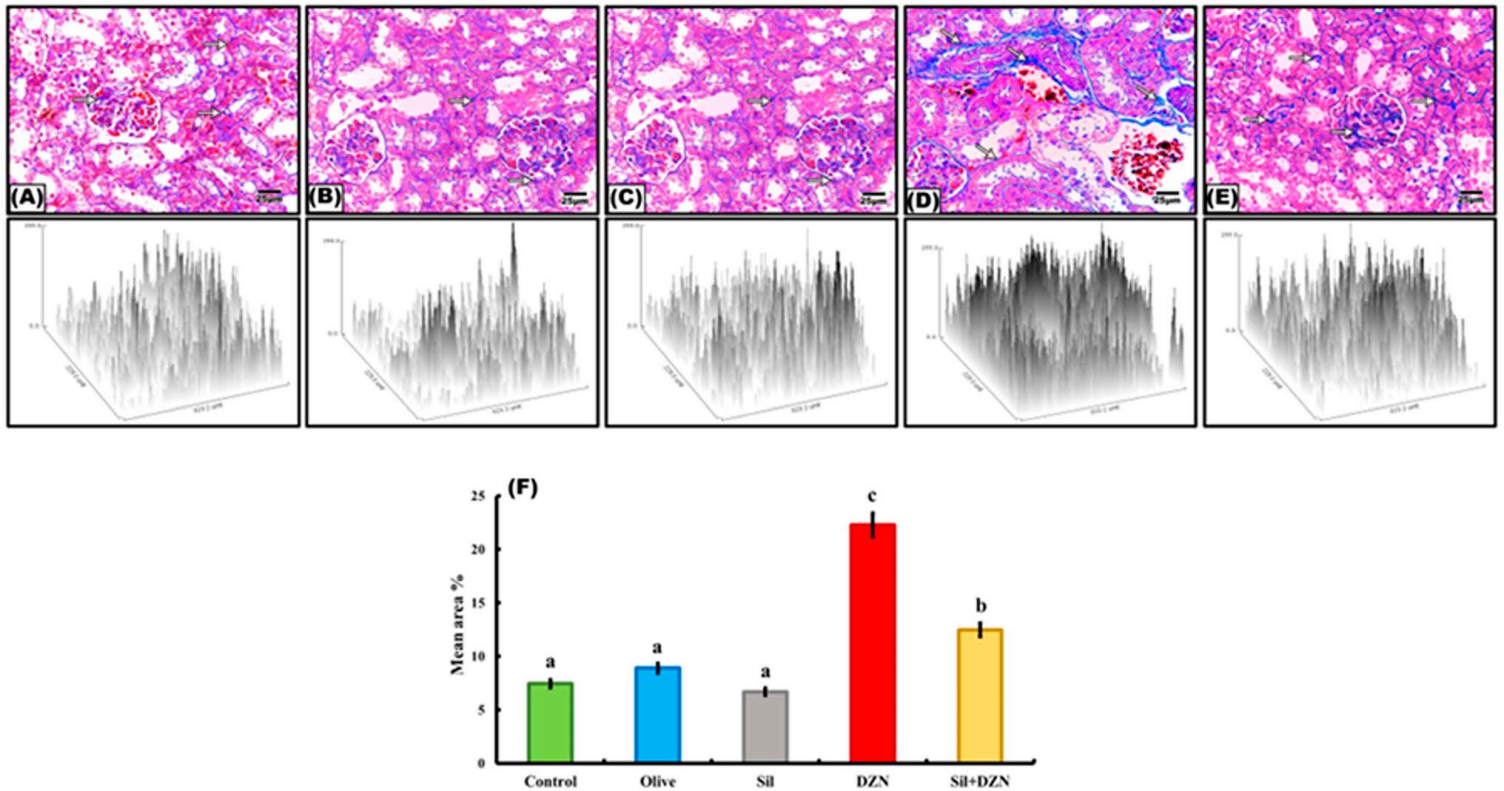
Photomicrographs of kidney sections stained with Masson’s trichrome. **A**–**C** Normal control rats (G1), olive oil-treated rats (G2) and rats treated with silymarin (G3). The kidney showed minimal collagen fibers among the glomerular capillaries (arrow) and surrounding the renal corpuscles and tubules (arrow). **D** The kidneys of diazinon-intoxicated rats (G4) showed the accumulation of collagen fibers among the glomerular capillaries (arrow) and surrounding the renal corpuscles and tubules (arrow). **E** Diazinon-intoxicated rats treated with silymarin (G5) showed minimal collagen fibers among the glomerular capillaries (arrow). **F** Histogram representing the mean area percentage of collagen fiber deposition in all the experimental groups. The scale bar represents 25 μm

The examination of kidney sections stained with Masson’s trichrome showed that the intoxicated group (G4) displayed a significant increase in the amount of collagen fiber deposition between the tubules and among the glomerular capillaries (Fig. 3D). On the other hand, the rats in the intoxicated group which were treated with SIL (G5) exhibited decreased amounts of collagen fibers among the glomerular capillaries and surrounding the renal corpuscles and tubules (Fig. 3E). Minimal amounts of collagen fibers among the glomerular capillaries and surrounding the renal corpuscles and tubules were detected in the control, olive oil, and SIL-treated groups (Fig. 3A–C).

### Immunohistochemical assessment of kidney tissue

**Fig. 4:**
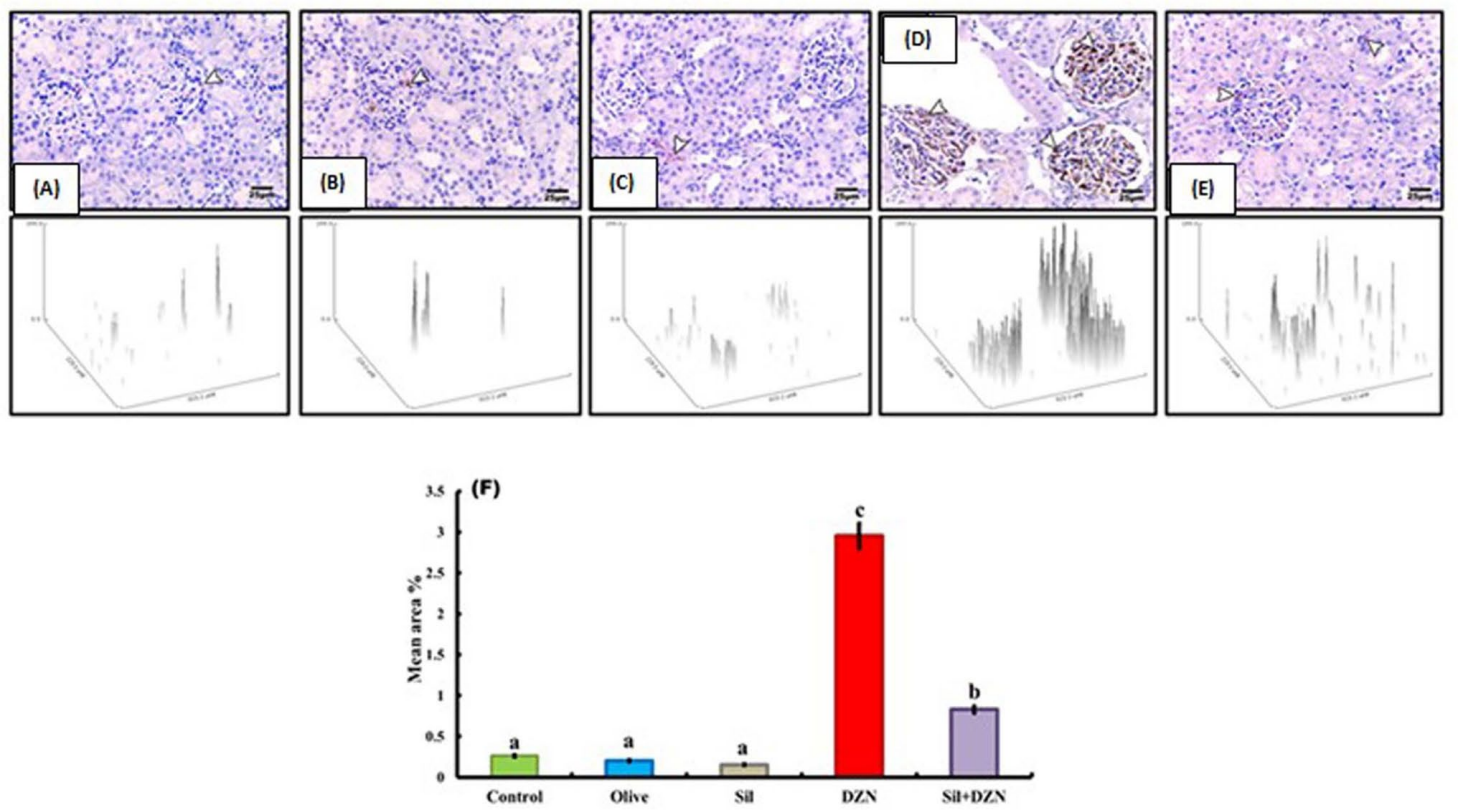
Tumor necrosis factor alpha (TNF-α) immunohistochemical staining of kidney sections from experimental rats. **A**–**C** Normal control rats (G1), olive oil-treated rats (G2) and rats treated with silymarin (G3) showed minimal changes in glomerular epithelial cells. **D** Diazinon-intoxicated rats (G4) showed a strong positive cytoplasmic reaction in glomerular epithelial cells (arrow). **E** After the rats were treated with diazinon and then with silymarin (G5), minimal positive cytoplasmic reactions were detected in the glomerular epithelial cells (arrow). **F** Histogram showing the mean area percentage of TNF-α-immunoreactive cells in all the experimental groups (anti-TNF-α, ×400). The scale bar represents 25 μm

**Fig. 5:**
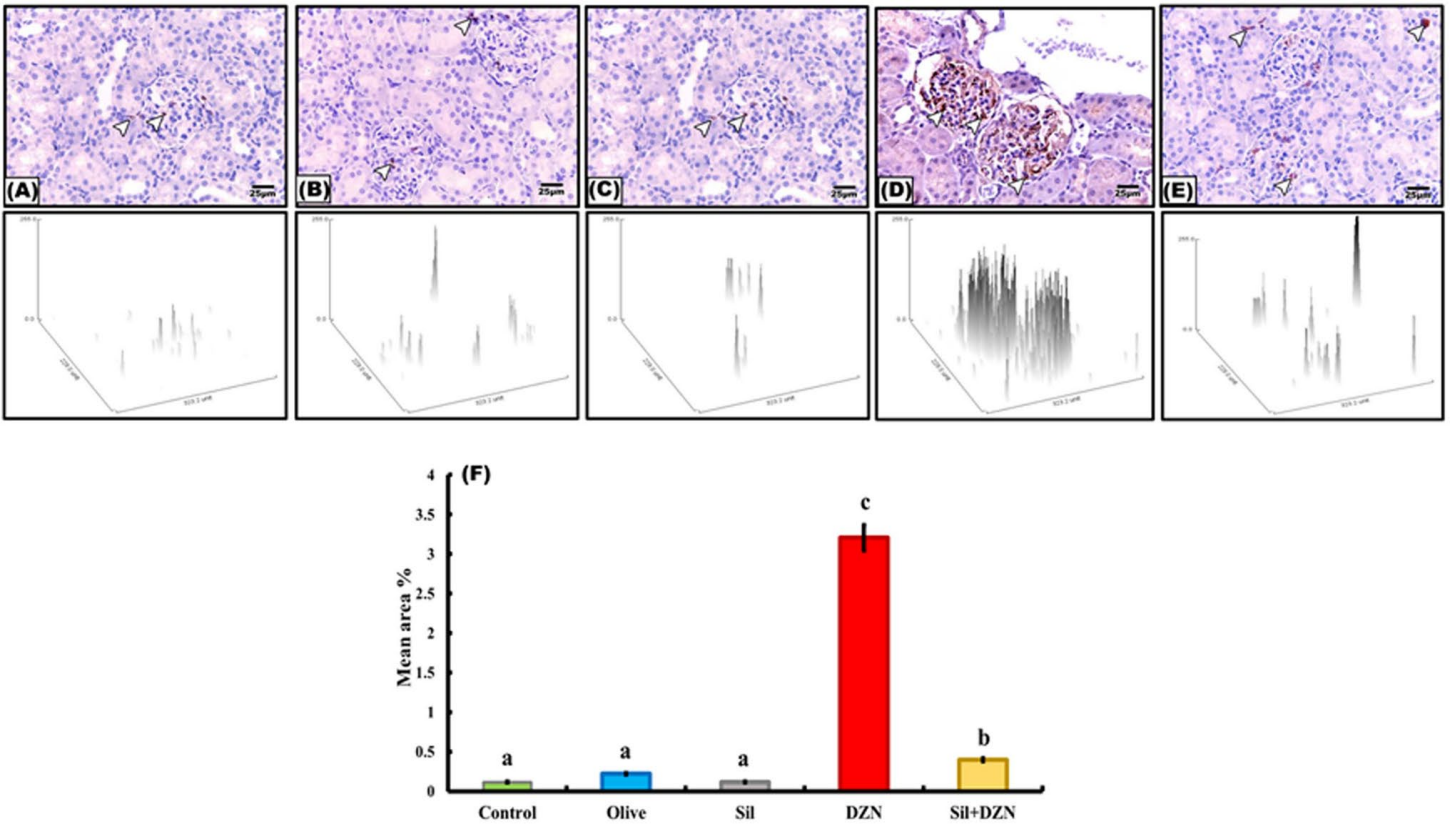
Cyclooxygenase 2 (COX2) immunohistochemical staining of kidney sections from experimental rats. **A**–**C** Normal control rats (G1), olive oil-treated rats (G2) and rats treated with silymarin (G3) showed minimal changes in glomerular epithelial cells. **D** Diazinon-intoxicated rats (G4) showed a strong positive cytoplasmic reaction in glomerular epithelial cells (arrow). **E** After the rats were treated with diazinon and then with silymarin (G5), minimal positive cytoplasmic reactions were detected in the glomerular epithelial cells (arrow). **F** Histogram representing the mean area percentage of COX2-immunoreactive cells in all the experimental groups (anti-COX2, × 400). The scale bar represents 25 μm

Tumor necrosis factor-α (TNF-α) and cyclooxygenase 2 (COX2) immunohistochemical staining of kidney tissue sections revealed that the glomerular epithelial cells of the intoxicated rats which were exposed to DZN (G4) had strong positive cytoplasmic signals (Figs. 4D, 5D). However, the glomerular epithelial cells of the intoxicated rats treated with SIL (G5) exhibited minimal positive cytoplasmic reactions (Figs. 4E, 5E). TNF-α and cyclooxygenase 2 (COX2) immunohistochemical staining of kidney tissues from control, olive oil-treated, and SIL-treated rats revealed minimal changes in glomerular epithelial cells (Figs. 4A–C, 5A–C).

### Morphometric study

The mean percentages of Masson’s trichrome-, TNF-α- and COX2-immunoreactive areas for all groups are presented in Figs. 3F, 4F, and 5F, respectively. Compared with those of the control, olive oil-treated, and SIL-treated rats, the mean area percentage of the DZN-treated (G4) group was significantly increased. However, intoxicated rats treated with SIL (G5) showed a significant decrease in the mean area percentage.

## Discussion

This study investigated the ability of SIL to maintain kidney function in Wistar rats administered DZN to induce subacute nephrotoxicity. The lack of adequate knowledge regarding the metabolism and carcinogenicity of agricultural pesticides, such as organophosphate pesticides, as well as the need for a medication or medical herb to lessen the negative effects of their metabolites, appear to be major human health concerns [22]. In the present study, no evident clinical indications of DZN at a dose of 15 mg/kg b.wt. were detected, which revealed that no signs of stress were present in the rats.

This study revealed significant decreases in the RBC count, Hb content, and HCT value, together with a lack of significant differences in the MCH and MCHC values, suggesting normocytic hypochromic anemia in the group of rats that received DZN. Treatment of intoxicated rats with SIL significantly increased the RBC count, Hb content, and HCT. This effect may be attributed to decreased free radical generation in the erythrocyte membrane [23]. The DZN-treated group showed a significant increase in WBC counts with a significant decrease in the platelet number. The increased number of WBCs may be attributed to tissue damage and toxicity caused by DZN, which activates defense mechanisms of the immune system [24], and these results agree with those of Salehzadeh et al. [25]. The significant decrease in the platelet count in the DZN-intoxicated group may be due to the effects of free radicals on the bone marrow that depress thrombopoiesis [26], and these results agree with those reported by Elsharkawy et al. [27].

In this study, a significant increase in the serum urea and creatinine levels was observed in the DZN-intoxicated group at 4 weeks, which indicated kidney injury. The elevated serum urea and creatinine levels may have been due to impaired renal function, which is characterized by a rapid reduction in the ability of the kidney to eliminate waste products, resulting in the accumulation of the normal end products of nitrogen metabolism either from the diet or normal tissue catabolism and a corresponding increase in urea and creatinine levels [28]. A similar increase in urea and creatinine levels caused by DZN was reported in a previous study [29]. A significant decrease in the serum urea and creatinine levels was observed when rats were cotreated with DZN and SIL. This result might have been due to the nephroprotective activity of SIL [30].

The present study revealed that the MDA content markedly increased, while the CAT, SOD, GPx, and GSH levels decreased significantly after 4 weeks of DZN treatment, which indicated oxidative stress. The elevated levels of MDA and decreased levels of CAT, SOD, GPx, and GSH may have been due to DZN inducing tissue damage by increasing lipid peroxidation byproducts and decreasing the level of antioxidants [31]. These findings were confirmed by the improvement in images of Masson’s trichrome staining of the kidneys of DZN-intoxicated rats, which revealed an increase in collagen fiber deposition between the tubules and within the glomerular capillaries. These findings show how ROS contribute to DZN-mediated nephrotoxicity and damage [32]. On the other hand, a significant decrease in the MDA level and significant increases in the levels of CAT, SOD, GPx, and GSH were observed in the rats treated with SIL. SIL has antioxidant potential, and these findings agree with Abouzeinab [33], who observed the antioxidant effects of SIL on nephropathy.

In the present study, a significant increase in the IL-1β and IL-6 levels was observed in the DZN-intoxicated group at the end of 4 weeks, which indicated kidney injury. IL-1β is a potent inflammatory cytokine produced mainly by macrophages [34]. IL-1β, either directly or through the induction of other cytokines such as IL-6 and TNF-α, promotes the development of kidney inflammation by increasing the expression of adhesion molecules and chemotactic factors and by recruiting inflammatory cells, thereby inducing kidney injury [35]. The presence of SIL attenuates the proinflammatory effects of DZN by downregulating IL-1β and IL-6 expression. Yang et al. [36] reported the anti-inflammatory potential of SIL through the inhibition of IL-1β and IL-6 expression. These findings confirmed the improvement in TNF-α and COX2 immunohistochemical staining in the kidneys of DZN-treated rats, which showed strong positive cytoplasmic reactions in glomerular epithelial cells. However, the glomerular epithelial cells of the intoxicated rats treated with SIL exhibited minimal positive cytoplasmic reactions. These findings coincided with those reported by Vangaveti et al. [37].

The present study revealed a marked increase in the level of the NF-κB protein marker and a marked decrease in the levels of the Keap-1, HO-1, and Nrf2 protein markers in kidney tissue, which was confirmed by the significant upregulation of the NF-κB mRNA and significant downregulation of the Keap-1, HO-1, and Nrf2 mRNAs in the kidneys of the intoxicated group after 4 weeks of DZN treatment; these findings may indicate pyroptosis-mediated cell death. Pyroptosis is considered a type of programmed inflammatory cell death that elaborates on the pathogenesis of acute kidney injury [38]. Exposure to DZN induced ROS generation and decreased the activity of mitochondrial antioxidant enzymes, which led to the activation of the NF-κB pathway, thereby inducing kidney injury [39]. On the other hand, the intoxicated group treated with SIL showed significant decreases in the level of NF-κB, with significant increases in the levels of Keap-1, HO-1, and Nrf2 protein markers in kidney tissue and significant downregulation of the NF-κB mRNA, with significant upregulation of Keap-1, HO-1, and Nrf2 mRNA expression in the kidney. These findings are attributed to the anti-inflammatory effects of SIL via the inhibition of the NF-κB pathway [40]. On the other hand, the Nrf2–Keap-1 pathway protects kidneys from the harmful effects of oxidative stress and is essential for preventing DZN-induced toxicity [41]. These findings agree with those of Zare et al. [42].

These findings confirmed the improvement in the histopathological images of the kidneys from SIL-treated rats compared with the kidneys from DZN-intoxicated rats, which showed a dilated Bowman’s space, inflammatory cell infiltration, glomerular and peritubular vascular congestion and swelling of renal tubular epithelium cells. These findings coincided with those of previous research [43]. However, treatment of intoxicated rats with SIL decreased Bowman’s space dilatation, inflammatory cell infiltration, glomerular and peritubular vascular congestion and renal tubular epithelial cell swelling. These findings coincided with those of Ustyol et al. [44]. Therefore, SIL can reduce oxidative stress by directly scavenging free radicals, inhibiting certain enzymes that produce free radicals, preserving the integrity of the electron transport chain, promoting an optimal cellular redox status, enhancing the activity of enzymes in the endogenous antioxidant system, inducing nonenzymatic antioxidant defenses such as glutathione or transcription factors (Nrf2 and NF-κB), and inducing the transcription of specific genes [45].

## Conclusions

As a potential nephroprotective drug against DZN-induced nephrotoxicity, SIL was highlighted in the current investigation. As SIL increased kidney CAT, SOD, GPx, and GSH antioxidant levels and inhibited the production of TNF-α and COX2 in renal tissues through the inhibition of the NF-κB pathway, it protected the kidney from DZN insult and decreased lipid peroxidation by lowering kidney MDA levels. The ability of silymarin to combat oxidative stress may be related to its modification of the Keap1/Nrf2 pathway and subsequent overexpression of downstream antioxidant enzymes such as CAT, SOD, GPx, and GSH. Therefore, this research might allow SIL to be used in the future as a nephroprotective drug, increasing its medical usefulness.

## Supplementary Information

Below is the link to the electronic supplementary material.

Supplementary file1 (DOCX 18 KB)

Supplementary file2 (JPG 115 KB)

Supplementary file3 (JPG 79 KB)

Supplementary file4 (JPG 514 KB)

## Data Availability

The corresponding author will deliver the information needed to back up this study’s conclusions upon request.
