# Supplementary material for: Silymarin ameliorates diazinon-induced subacute nephrotoxicity in rats via the Keap1–Nrf2/heme oxygenase-1 signaling pathway
Source: Forensic Toxicol. 2024 Aug 8;43(1):62–73. doi: 10.1007/s11419-024-00697-x (PMC11782450; doi:10.1007/s11419-024-00697-x)
Supplement: Supplementary file 1 — Supplementary file1 (DOCX 18 KB) [file 11419_2024_697_MOESM1_ESM.docx]

**Table 1** The evaluated genes’ primer sets.

| Gene symbol | Forward | Reverse | Gene bank |
| --- | --- | --- | --- |
| NFκB | TCTGTTTCCCCTCATCTTTCC | GCGTCTTAGTGGTATCTGTGCTT | AF079314.2 |
| Keap-1 | GGAATGCTATGACCCAGACA | TGCTCAGGTAGTCCAAGTGC | XM_032909789.1 |
| HO-1 | CGTGCAGAGAATTCTGAGTTC | AGACGCTTTACGTAGTGCTG | NM 012580 |
| Nrf2 | GCAACTCCAGAAGGAACAGG | AGGCATCTTGTTTGGGAATG | NM_031789.2 |
| GAPDH | CACCCTGTTGCTGTAGCCATATTC | ACATCAAGAAGGTGGTGAAGCAG | XM_032910454.1 |

Table 2 Effect of silymarin versus diazinon on complete blood cell count in the experimental groups of rats after 4 weeks of treatment (mean±standard error, n=6).

| **Tested Parameters** | **Groups** | | | | |
| --- | --- | --- | --- | --- | --- |
|  | Control | Olive oil | Silymarin | Diazinon | Silymarin+Diazinon |
| RBCs (×10^6^/µl) | 7.66±0.21**^bc^** | 8.00±0.21**^c^** | 7.32±0.27**^bc^** | 6.49±0.13**^a^** | 6.88±0.18**^ab^** |
| Hb (g/dl) | 12.83±0.47**^c^** | 12.80±0.46**^c^** | 11.94±0.28**^bc^** | 10.44±0.18**^a^** | 11.32±0.34**^ab^** |
| HCT (%) | 40.18±1.21**^c^** | 39.84±1.18**^bc^** | 38.17±0.84**^bc^** | 34.36±0.46**^a^** | 36.84±0.74**^ab^** |
| MCV (fl) | 51.93±1.15**^a^** | 49.00±0.84**^a^** | 51.92±1.09**^ab^** | 51.92±0.84**^ab^** | 53.14±0.60**^b^** |
| MCH (pg) | 16.56±0.51**^a^** | 15.21±0.27**^a^** | 16.37±0.37**^a^** | 15.76±0.19**^a^** | 16.56±0.24**^a^** |
| MCHC (g/dl) | 31.88±0.36**^a^** | 30.58±0.15**^a^** | 31.52±0.14**^a^** | 30.40±0.23**^a^** | 31.12±0.12**^a^** |
| PLT (×10^3^/µl) | 897.66±55.24**^b^** | 908.28±50.83**^b^** | 907.25±78.20**^b^** | 602.00±77.15**^a^** | 840.40±41.83**^b^** |
| WBCs (×10^3^/µl) | 6.92±0.33**^a^** | 7.55±0.88**^ab^** | 8.68±0.20**^ab^** | 14.78±1.06**^c^** | 9.84±1.12**^b^** |
| Neutrophils (×10^3^/µl) | 2.20±0.28**^a^** | 2.45±0.50**^a^** | 2.25±0.20**^a^** | 5.26±0.94**^b^** | 2.94±0.32**^a^** |
| Lymphocytes (×10^3^/µl) | 4.26±0.32**^a^** | 4.64±0.54**^ab^** | 6.11±0.87**^c^** | 8.22±0.47**^c^** | 6.04±0.35**^bc^** |
| Monocytes (×10^3^/µl) | 0.23±0.10**^a^** | 0.34±0.03**^a^** | 0.27±0.10**^a^** | 1.14±0.49**^b^** | 0.28±0.27**^a^** |
| Eosinophils (×10^3^/µl) | 0.05±0.03**^a^** | 0.11±0.02**^a^** | 0.05±0.02**^a^** | 0.50±0.22**^b^** | 0.16±0.10**^a^** |
| Basophils (×10^3^/µl) | 0.06±0.04**^a^** | 0.10±0.03**^a^** | 0.10±0.05**^a^** | 0.15±0.08**^a^** | 0.08±0.07**^a^** |

Means with different superscripts in the same row are significantly different at p<0.05.

Table 3 Effect of silymarin versus diazinon on oxidative markers in the kidney homogenates in the experimental groups of rats after 4 weeks of treatment (mean±standard error, n=6).

| **Tested Parameters** | **Groups** | | | | |
| --- | --- | --- | --- | --- | --- |
|  | Control | Olive oil | Silymarin | Diazinon | Silymarin+Diazinon |
| CAT  nmol/mg protein | 2.44±0.18^b^ | 2.60±0.19^b^ | 2.50±0.10^b^ | 1.40±0.09^a^ | 2.48±0.17^b^ |
| SOD  U/mg protein | 3.08±0.17^b^ | 2.92±0.31^b^ | 3.18±0.40^b^ | 1.23±0.03^a^ | 2.70±0.19^b^ |
| GPx  nmol/mg protein | 24.00±1.25^b^ | 22.62±1.50^b^ | 22.44±0.91^b^ | 7.28±0.52^a^ | 21.40±1.87^b^ |
| GSH  µg/mg protein | 1.64±0.06^c^ | 1.66±0.08^c^ | 1.68±0.07^c^ | 0.45±0.03^a^ | 1.44±0.09^b^ |
| MDA  nmol/mg protein | 0.33±0.04^a^ | 0.37±0.04^a^ | 0.43±0.06^a^ | 2.25±0.05^c^ | 0.79±0.02^b^ |

Means with different superscripts in the same row are significantly different at p<0.05.
