# Supplementary figures and images for: Silymarin ameliorates diazinon-induced subacute nephrotoxicity in rats via the Keap1–Nrf2/heme oxygenase-1 signaling pathway
Source: Forensic Toxicol. 2024 Aug 8;43(1):62–73. doi: 10.1007/s11419-024-00697-x (PMC11782450; doi:10.1007/s11419-024-00697-x)

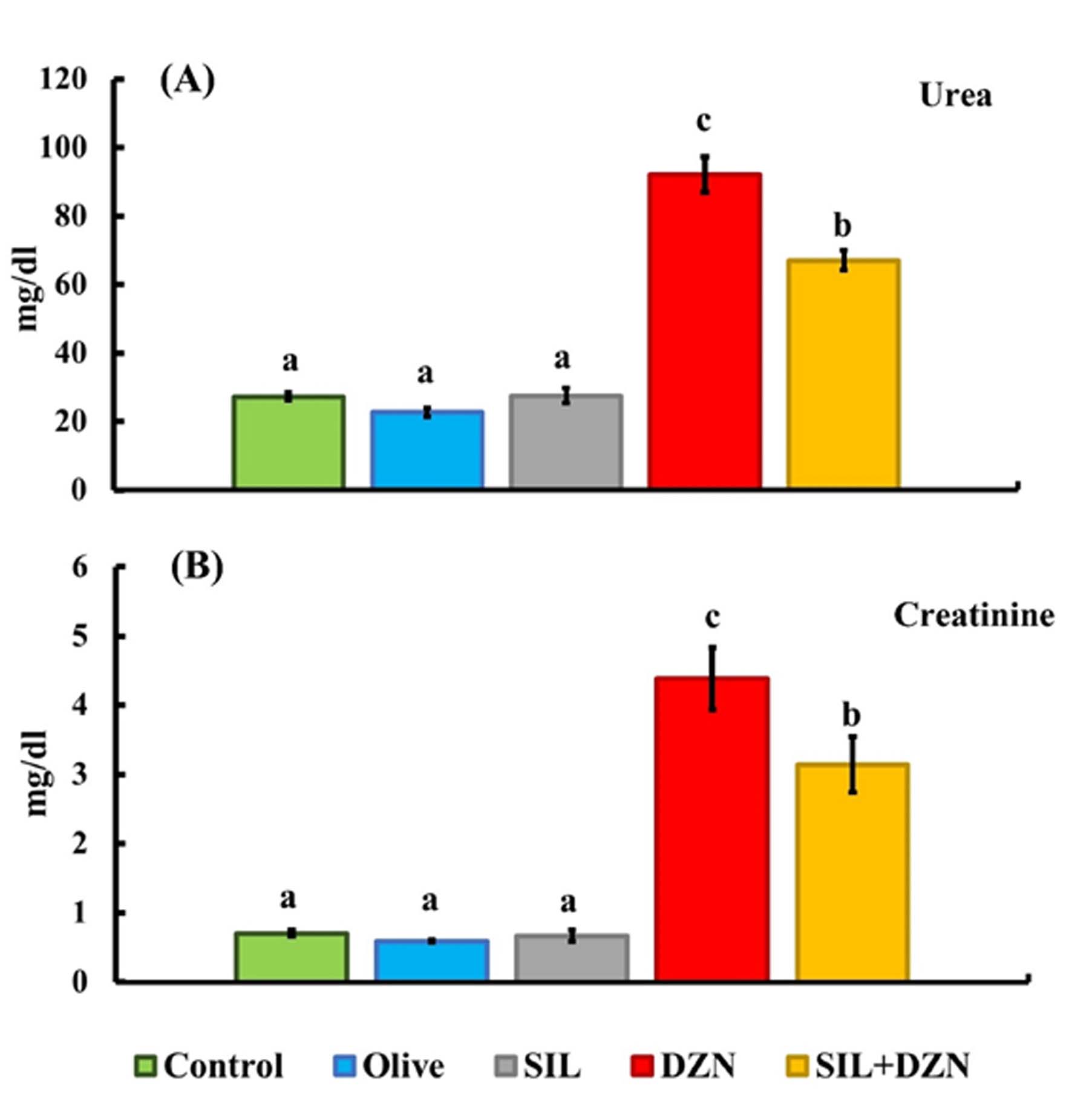

Supplement: Supplementary file 2 — Supplementary file2 (JPG 115 KB) [file 11419_2024_697_MOESM2_ESM.jpg]

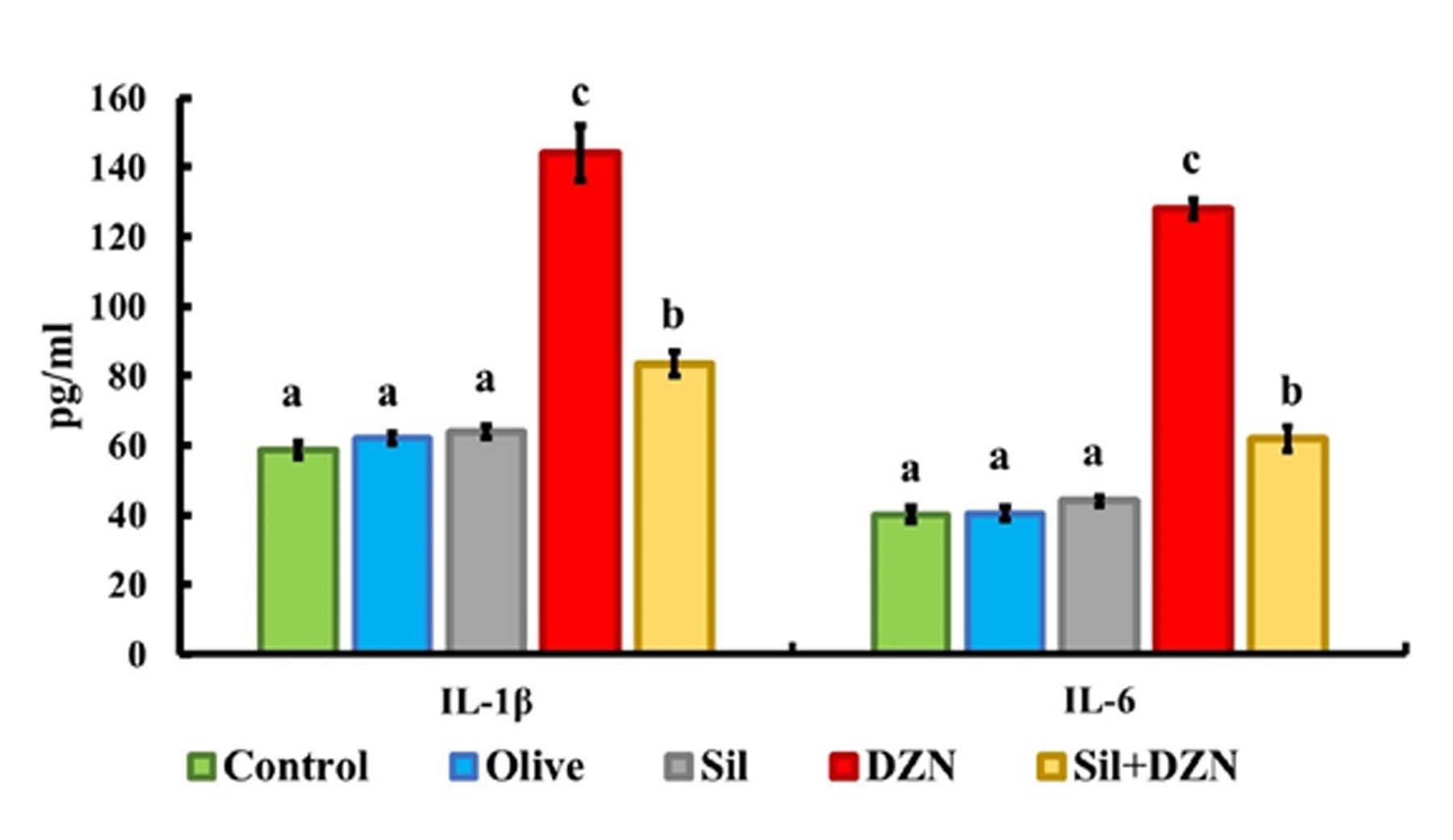

Supplement: Supplementary file 3 — Supplementary file3 (JPG 79 KB) [file 11419_2024_697_MOESM3_ESM.jpg]

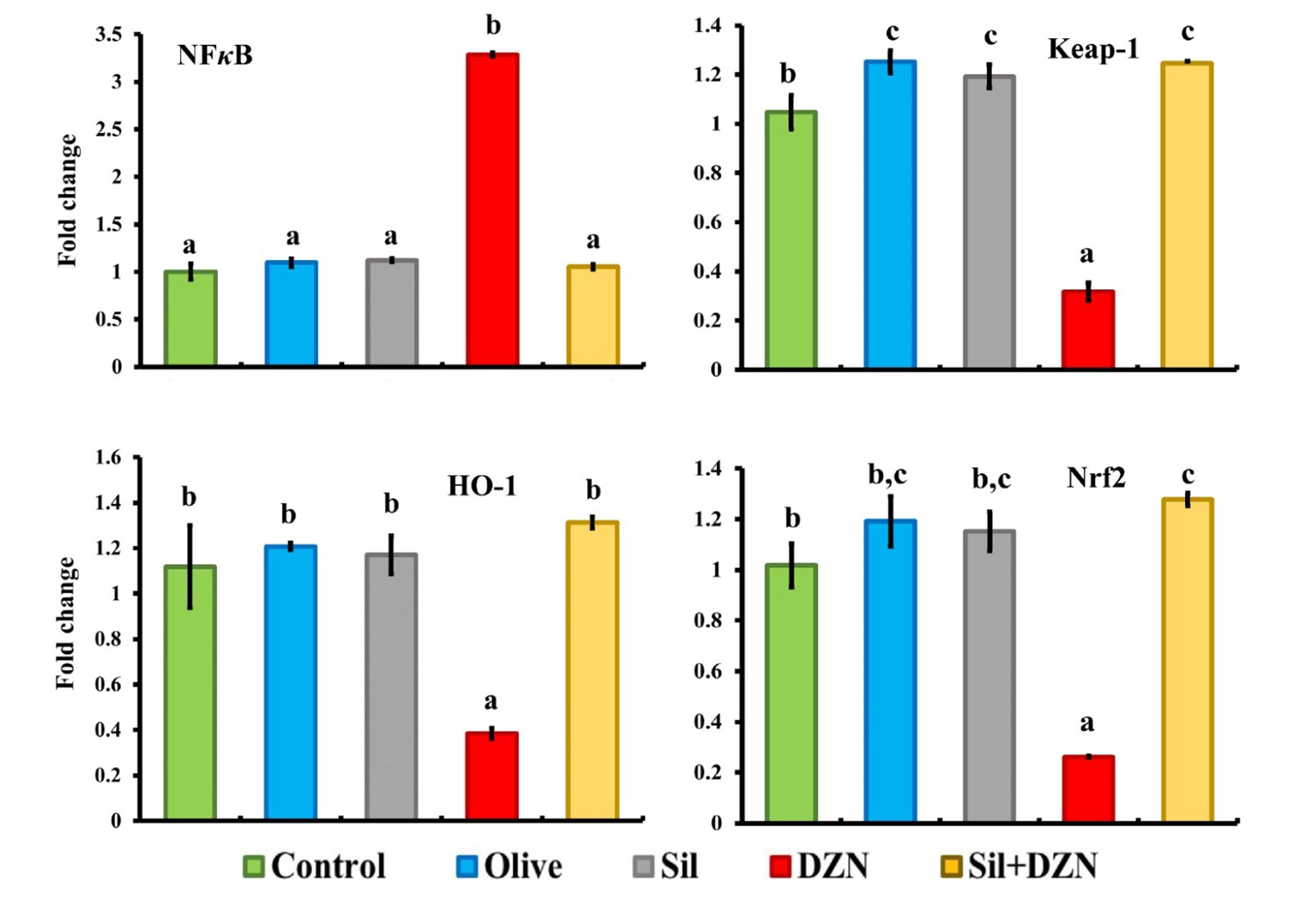

Supplement: Supplementary file 4 — Supplementary file4 (JPG 514 KB) [file 11419_2024_697_MOESM4_ESM.jpg]
